# Effects of the Glutamine Administration on T Helper Cell Regulation and Inflammatory Response in Obese Mice Complicated with Polymicrobial Sepsis

**DOI:** 10.1155/2020/8869017

**Published:** 2020-11-10

**Authors:** Chiu-Li Yeh, Li-Han Su, Jin-Ming Wu, Po-Jen Yang, Po-Chu Lee, Po-Da Chen, Chun-Chieh Huang, Der-Yirng Hsieh, Hsueh-Ju Wang, Sung-Ling Yeh, Ming-Tsan Lin

**Affiliations:** ^1^School of Nutrition and Health Sciences, College of Nutrition, Taipei Medical University, Taipei, Taiwan; ^2^Department of Surgery, National Taiwan University Hospital and College of Medicine, National Taiwan University, Taipei, Taiwan; ^3^Department of Surgery, National Taiwan University Hospital Hsin-Chu Biomedical Science Park Branch, Hsin-Chu County, Taipei, Taiwan; ^4^Department of Surgery, National Taiwan University Hospital Hsin-Chu Branch, Hsin-Chu County, Taiwan; ^5^Department of Nursing, Nutrition Support Team, National Taiwan University Hospital, Taipei, Taiwan; ^6^Department of Pharmacy, National Taiwan University Hospital, Taipei, Taiwan

## Abstract

This study investigated the impacts of GLN on inflammation and T cell dysregulation in obese mice complicated with sepsis. Mice were divided into normal control (NC) and high-fat diet groups. The high-fat diet provided 60% of energy from fat and was administered for 10 weeks to induce obesity. Mice fed with a high-fat diet were then assigned to sham (SH) and sepsis with saline (SS) or GLN (SG) groups. The SH group was subjected to laparotomy, while the sepsis group underwent cecal ligation and puncture (CLP). The SS group was intravenously injected with saline. The SG group was intravenously administered GLN after CLP. Mice were sacrificed at 12, 24, or 48 h post-CLP, respectively. Results demonstrated that in the presence of obesity, sepsis drove CD4+ T cells toward the helper T (Th)2 and Th17 lineages. Also, expressions of inflammatory cytokines and macrophage infiltration markers in adipose tissues and lungs were elevated. Treatment of obese mice with GLN after sepsis reversed Th polarization and downregulated macrophage infiltration and inflammatory cytokine, whereas the tight junction-associated protein expression increased in the lungs. These findings suggest that the intravenous administration of GLN to obese mice after sepsis modulated a more balanced Th cell lineage, alleviated inflammation, and attenuated lung injury.

## 1. Introduction

Obesity is a complex, multifactorial condition that has become an important global health problem. Excessive adipose tissue accumulation was shown to be associated with low-grade inflammation and endothelial dysfunction which may consequently result in cardiovascular diseases, diabetes, stroke, etc. [1]. Also, obese subjects were found to have dysregulated innate and adaptive immune responses that may worsen disease outcomes [1, 2]. Obese patients are considered physiologically frail, and they may experience postinfection complications and increased mortality in the long-term [2]. A previous study showed that obesity is associated with an increased risk of nosocomial and secondary infections that lead to sepsis [3].

Sepsis is a life-threatening syndrome with multiorgan dysfunction [4]. Decline of T lymphocytes and impairment of the T cell function are characteristics of sepsis [5, 6]. The dysregulation of T lymphocytes results in an imbalance between pro- and anti-inflammatory reactions that aggravates organ damage and subsequent outcomes [7]. Because the prevalence of obesity has reached epidemic proportions worldwide, obesity has become a cause of concern in septic patients. A former study showed that the inflammatory response is exaggerated in obese subjects compared to their lean counterparts during sepsis [8]. Although some studies indicated that obesity is associated with lower mortality in the critically ill compared to patients with a normal weight [9–11], one clinical study reported that upon intensive care unit (ICU) admission, obese patients had higher mortality among sepsis patients than those with a normal weight [12]. Such discrepancies can possibly be explained by patient characteristics and limitations of a confounded interpretation. Previous reports concerned with the dysregulation of T cells exclusively emphasized obesity or sepsis. Studies evaluating the effects of obesity on T cell polarization during sepsis are rare, and those effects need to be investigated.

Glutamine (GLN) is an amino acid with immunomodulatory properties. Previous studies found that the GLN administration attenuates inflammatory responses and has favorable effects on balancing T cell dysregulation and abnormal metabolic functions in sepsis [13–16]. Although GLN seems to have beneficial effects on sepsis, the physiological alterations of comorbid obesity and sepsis differ from exclusive sepsis. We are unaware of any study investigating the impacts of GLN on T cell regulation and subsequent organ injury in obese subjects complicated with sepsis. In this study, mice were fed a high-fat diet to create a condition of obesity, and cecal ligation and puncture (CLP) was subsequently performed to induce peritonitis. CLP is a well-established rodent model in sepsis and mimics the pathological alterations in human septic patients [17]. Since a cluster of differentiation 4-positive (CD4+) T helper (Th) cells are important lymphocyte subsets that influence innate and adaptive immunity during sepsis [18], the impacts of the parenteral GLN administration after peritonitis were investigated. We hypothesized that GLN may have favorable effects on regulating CD4+ T cell, alleviating inflammatory response, and subsequent organ injury in obesity complicated with sepsis.

## 2. Materials and Methods

### 2.1. Animals

In this study, 5-week-old male C57BL/6 mice (weighing around 20 g) were used. All mice were acclimated in the Laboratory Animal Center at Taipei Medical University (TMU; Taipei, Taiwan). The conditions of the Animal Center were a temperature of 21 ± 2°C and relative humidity of 50% ~55% with a 12-h light-dark cycle. During the acclimation period, water and standard rodent chow diet (Purina no. 5001, Fort Worth, TX, USA) were provided ad libitum. Protocols of the experiment were approved by the Animal Care and Use Committee of TMU. Care and use of laboratory animals were in compliance with the *Guide for the Care and Use of Laboratory Animals* (National Research Council, 1996).

### 2.2. Experimental Procedures

Mice were randomly assigned to a normal control (NC, *n* = 6) group and a high-fat group (HF, *n* = 78). Mice in the NC group were fed a standard rodent chow diet, while mice in the HF group were provided a diet with 60% kcal as fat for 10 weeks [19]. The high-fat diet composition is shown in Table 1 as provided by the commercial company (Research Diets, New Brunswick, NJ, USA). At the end of the 10-week experimental period, several mice in the HF group (*n* = 6) were sacrificed and treated as the positive control for the NC group. The remaining mice in the HF group were subdivided into sham (SH, *n* = 24), sepsis+saline (SS, *n* = 24), and sepsis+glutamine (SG, *n* = 24) groups. Mice in the sham group were subjected to a laparotomy without CLP, while the SS and SG groups were subjected to CLP as previously described [17]. Mice were given an intraperitoneal injection of zoletil (25 mg/kg body weight (BW), Virbac, Carros, France), and rompun (10 mg/kg BW), Bayer, Leverkusen, Germany) for anesthetization. Then, a 1 cm incision was made on the abdominal wall to open the peritoneum. The cecum was ligated at 50% below the ileocecal valve, and the cecum was punctured through with a 23-gauge needle. A small drop of feces was squeezed out and smeared onto the abdomen. A continuous suturing technique was performed to close the incision. The CLP surgery was performed by the same person in all animals to ensure consistency. After the surgery, animals were subcutaneously rehydrated with sterile saline (40 mL/kg BW), and all mice were allowed free access to water and rodent chow. Postoperative pain was managed by treatment with 100 *μ*L of 0.25% bupivacaine which was administered at the incision site before skin closure. Mice were sacrificed at 12, 24, or 48 h after CLP according to the respective grouping. Mice scheduled for sacrifice at 12 and 24 h post-CLP were intravenously injected with a single dose of either saline or GLN (0.75 g GLN/kg BW) via a tail vein 1 h after CLP while mice sacrificed at 48 h were injected with another dose of the same treatment 24 h after the first dose. GLN was administered as alanyl-glutamine dipeptide (Dipeptiven; Fresenius-Kabi, Homburg, Germany). This dosage was proven to have immunoregulatory effects during sepsis [20, 21]. Mice were anesthetized with ether and euthanized by cardiac puncture. Blood samples were collected. Part of the fresh whole blood was used to analyze the percentage of the CD4^+^ T cell subpopulation. Remaining blood samples were centrifuged at 700 × *g* and 4°C for 15 min to obtain plasma. The peritoneum was opened and irrigated with 5 mL/100 g BW of saline to obtain the peritoneal lavage fluid (PLF). The plasma and PLF were stored at −80°C. Epididymal tissues were weighed, and the lungs were excised. Tissues were frozen in liquid nitrogen and stored at -80°C for further analysis.

### 2.3. Measurements of the CD4^+^ T Cell Subpopulation

The measured CD4^+^ T cell subsets included Th1, Th2, Th17, and regulatory T (Treg) cells. Red blood cells (RBCs) were incubated with RBC Lysis Buffer (BioLegend, San Diego, CA, USA) for 15 min and washed in Dulbecco's Modified Eagle Medium (DMEM; Gibco, Waltham, MA, USA) and staining buffer (2% bovine serum albumin in phosphate-buffered saline), then resuspended in staining buffer. Whole blood (50 *μ*L) was stained according to the instruction manual. Intracellular staining for Th cells was performed using Intracellular Staining Permeabilization Wash Buffer and Fixation Buffer (BioLegend). Foxp3/Transcription Factor Staining Buffer (Invitrogen, Carlsbad, CA, USA) was used for Treg cells. Distributions of CD4^+^ T cell subpopuulations were determined by a flow cytometric analysis according to standard settings on a BD FACS-Canto II flow cytometer (BD Biosciences, San Diego, CA, USA). Data were analyzed with BD FACSDiva™ Software v. 8.0 (BD Biosciences). Antibodies used for intracellular cytokine staining were as follows: Pacific blue anti-CD3 (17A2, BioLegend), PerCP anti-CD4 (GK1.5, BioLegend), allophycocyanin (APC) anti-interferon (IFN)-*γ* (XMG1.2, BioLegend), phycoerythrin (PE) anti-IL4 (11B11, BioLegend), and fluorescein isothiocyanate (FITC) anti-IL17A (TC11-18H10.1, BioLegend). Percentages of Treg cells were analyzed by intracellular staining using APC anti-CD4 (GK1.5, BioLegend), PE anti-CD25 (3C7, BioLegend), and FITC-Foxp3 antibodies (150D, BioLegend). Ten thousand cells from the lymphocyte population were gated for Th1 (CD3^+^/CD4^+^/IFN-*γ*^+^), Th2 (CD3^+^/CD4^+^/IL-4^+^), Th17 (CD3^+^/CD4^+^/IL-17A^+^), and Treg cells (CD4^+^/CD25^+^/FoxP3^+^) during data analysis.

### 2.4. Measurements of Plasma Concentrations of Adipokines

Leptin and adiponectin were measured using enzyme-linked immunosorbent assay (ELISA) kits (R&D Systems, Minneapolis, MN, USA). The analytical protocols followed the instructions of the manufacturer.

### 2.5. Inflammatory Cytokine Concentrations in PLF

Interleukin (IL)-1*β* and IL-6 were measured by an ELISA in a microtiter plate. Antibodies specific to mouse IL-1*β* and IL-6 were first coated onto wells of microtiter strips, and then samples were added and developed with reagents (eBioscience, San Diego, CA, USA). The absorbance of each well was measured with a spectrophotometer.

### 2.6. Messenger (m)RNA Extraction and Analysis of a Real-Time Reverse-Transcription (RT) Quantitative Polymerase Chain Reaction (qPCR)

Lungs and epididymal tissues were homogenized, and the Trizol reagent (Invitrogen, Carlsbad, CA, USA) method was used to isolate total RNA. RNA pellets were dissolved in RNase-free water and stored at -80°C for further analysis. RNA concentrations were quantified by measuring absorbances at 260 and 280 nm on a spectrophotometer. A RevertAid™ first-strand complementary (c)DNA synthesis kit (Fermentas, Vilnius, Lithuania) was used to synthesize cDNA from total RNA. RT was performed by subsequent incubation for 5 min at 65°C, 60 min at 42°C, and 5 min at 70°C. cDNA was stored at -80°C until being used. Measured mRNA genes were amplified by a real-time RT-PCR using the 7300 Real-Time PCR System (Applied Biosystems, Foster City, CA, USA) with SYBR Green I as the detection format. Genes analyzed in adipose tissues included inflammatory cytokines (IL-1*β*, IL-6, and tumor necrosis factor (TNF)-*α*) and macrophage infiltration markers (CD68 and epidermal growth factor-like module-containing mucin-like hormone receptor-like (EMR)-1). Genes measured in lung tissues included macrophage infiltration markers, tight junction proteins (zonula occludens (ZO)-1 and occludin), and the antiapoptotic marker, B-cell lymphoma-extra large (Bcl-xL). Primers used in this study are described in Table 2. All primers were purchased from Mission Biotech (Taipei, Taiwan) based on deposited cDNA sequences (GenBank database, NCBI). Amplification was carried out in a total volume of 25 *μ*L containing a 1× Power SYBR Green PCR Master Mix (Applied Biosystems), 400 nM of each primer, and 100 ng of cDNA. The reaction was processed by one cycle of 2 min at 50°C and 10 min at 95°C, followed by 40 cycles of 15 s at 95°C and 1 min at 60°C, with a final dissociation curve (DC) analysis. Expression levels were quantified in duplicate by means of a real-time RT-PCR. The relative quantity of the mRNA expression was calculated by cycle threshold (CT) values which were normalized to mouse *β*-actin.

### 2.7. Statistical Analysis

All data are presented as the mean ± standarderror of the mean (SEM). Data were analyzed with the GraphPad Prism 5 statistical software program (GraphPad Software, La Jolla, CA, USA). Differences among groups at three different time points were analyzed by a one-way analysis of variance (ANOVA) followed by Tukey's post-hoc test. A *p* value of <0.05 was considered statistically significant.

## 3. Results

### 3.1. Changes in Tissue Weights and BWs and Plasma Adipokine Levels after High-Fat Diet Feeding

There were no differences in initial BWs between the NC and HF groups. After 10 weeks of feeding, the HF group had higher BWs than that of the NC group (NC 25.5 ± 0.6 g vs. HF 37.1 ± 0.9 g, *p* < 0.0001). Also, epididymal fat weights were higher in the HF group than the NC group (NC0.55 ± 0.03 g vs. HF 2.11 ± 0.13 g, *p* < 0.0001). Leptin concentrations in the HF group were significantly higher (NC 5.1 ± 3.2 ng/dl vs. HF 62.6 ± 3.9 ng/dl, *p* <0.0001), whereas adiponectin levels were lower than those of the NC group (NC 8.2 ± 0.4 mg/dl vs. HF 6.2 ± 0.2 mg/dl, *p* = 0.003).

### 3.2. Plasma Adipokine Levels in Obesity with Sepsis

**Figure 1:**
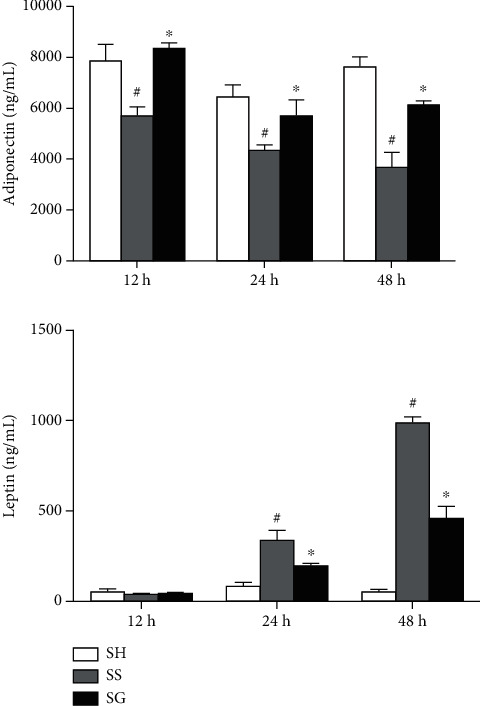
Plasma adiponectin and leptin concentrations in the obese sham-operated and sepsis groups at different time points. SH: sham-operated group; SS: sepsis group with saline; SG: sepsis group with glutamine. Values are expressed as the mean ± SEM. All data are representative of duplicate measurements at 12, 24, and 48 h after cecal ligation and puncture (CLP) (*n* = 8 for each respective group). Differences among groups at each time point were analyzed by a one-way ANOVA followed by Tukey's post-hoc test. The symbol “^#^” significantly differs from the SH group; the “^∗^” significantly differs from the SS group (*p* < 0.05).

Compared to the SH group, sepsis resulted in decreased plasma adiponectin levels, while leptin concentrations increased several fold post-CLP. The SG group had higher adiponectin, whereas leptin levels were lower than those of the SS group after CLP. There were no differences in adiponectin levels between the SH and SG groups at each time point post-CLP. Although no differences were noted at 12 and 24 h, the SG group had a higher leptin levels compared to the SH group at 48 h (Figure 1).

### 3.3. Blood CD4^+^ T Lymphocyte Populations in Obese Mice with Sepsis

**Figure 2:**
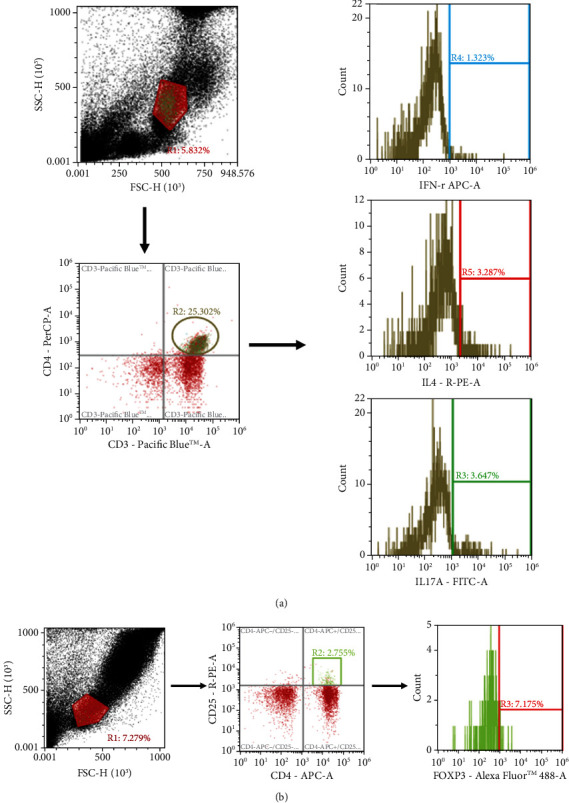
Representative flow cytometry data to illustrate the gating strategy for lymphocytes in blood. Lymphocytes were first identified based on low FSC and SSC characteristics. (a) CD4-positive (CD4+) lymphocytes were gated to analyze the percentages of IFN-*γ*-, IL-4-, and IL-17-expressing CD4+ cells. (b) Treg cells were identified as Foxp3^+^ CD25^+^ in CD4^+^ cells.

**Figure 3:**
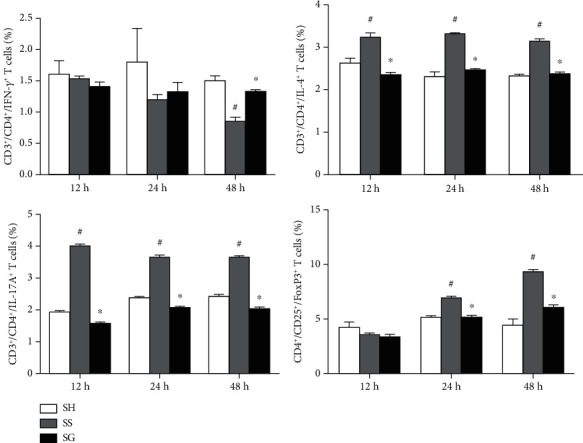
Percentages of helper T (Th) cell subpopulations in blood. Populations of Th cells were determined by CD4^+^ cells in CD3^+^ cells. As for subsets of Th cells, percentages of Th1 (interferon-*γ*-expressing cells), Th2 (interleukin (IL)-4-expressing cells), and Th17 cells (IL-17-expressing cells) among CD4^+^ lymphocytes were analyzed. Regulatory T (Treg) cells are presented as the percentage of CD25^+^Foxp3^+^ cells among CD4^+^ lymphocytes. SH: sham-operated group; SS: sepsis group with saline; SG: sepsis group with glutamine. Data are presented as the mean ± standarderror of the mean (SEM), *n* = 8 for each group. Differences in groups at each time point were analyzed by a one-way ANOVA with the Tukey's post-hoc test. The symbol “^#^” significantly differs from the SH group; the symbol “^∗^” significantly differs from the SS group (*p* < 0.05).

A typical flow cytometry chart was shown to illustrate the gating strategy for blood lymphocytes. CD4^+^ cells were gated to analyze the percentages of different cytokine-expressing CD4^+^ lymphocytes (Figure 2(a)), and Treg cells were identified as CD25^+^Foxp3^+^ in CD4^+^ cells (Figure 2(b)). Concerning subsets of CD4^+^ T cells, obesity with sepsis resulted in higher percentages of Th2 and Th17 cells at 12, 24, and 48 h and Treg cells at 24 and 48 h, while Th1-expressing cells had decreased by 48 h compared to the obesity sham group. The SG group exhibited a higher concentration of Th1 at 48 h and lower distributions of Th2 and Th17 at 12, 24, and 48 h and Treg at 24 and 48 h after CLP compared to the SS group. The Th1/Th2/Th17/Treg distributions in the SG groups did not differ from those of the obesity sham group at different time points (Figure 3).

### 3.4. PLF Cytokine Levels in Obese Mice with Sepsis

**Figure 4:**
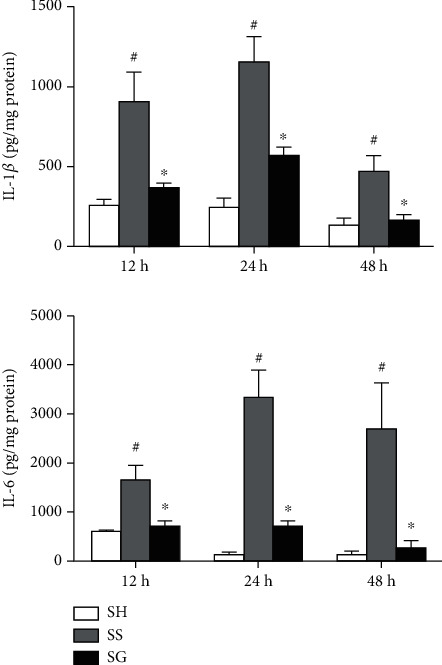
Concentrations of the inflammatory cytokines, interleukin (IL)-1*β*, and IL-6, in the peritoneal lavage fluid. SH: sham-operated group; SS: sepsis group with saline; SG: sepsis group with glutamine. Values are expressed as the mean ± SEM. All data are representative of duplicate measurements at 12, 24, and 48 h after cecal ligation and puncture (CLP) (*n* = 8foreachgroup). Differences among groups at each time point were analyzed by a one-way ANOVA followed by Tukey's post-hoc test. The symbol “^#^” significantly differs from the SH group; the symbol “^∗^” significantly differs from the SS group (*p* < 0.05).

In the presence of obesity, sepsis resulted in increased IL-1*β* and IL-6 levels in PLF. Compared to the SS group, the SG group exhibited lower IL-1*β* and IL-6 levels at each time point. Also, these cytokines in the SG group were higher than those detected from the SH group at 24 h after CLP (Figure 4).

### 3.5. mRNA Expressions of Adipose Tissue Macrophage Infiltration Markers and Inflammatory Mediators in Obese Mice with Sepsis

**Figure 5:**
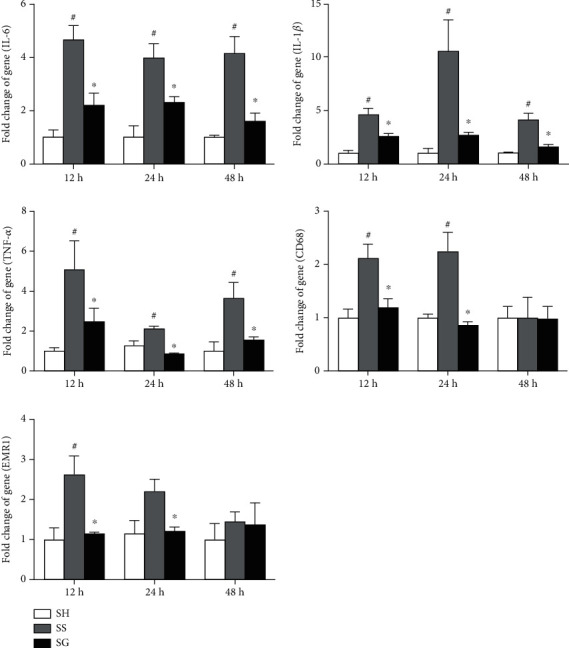
Messenger (m)RNA expressions of inflammatory cytokines and macrophage infiltration markers in epididymal fat tissues. IL-1*β*: interleukin-1*β*; IL-6: interleukin-6; TNF-*α*: tumor necrosis factor-*α*; cluster of differentiation (CD) 68; epidermal growth factor-like module-containing mucin-like hormone receptor-like 1 (EMR-1). SH: sham-operated group; SS: sepsis group with saline; SG: sepsis group with glutamine. mRNA changes were quantitated and analyzed by a real-time PCR and were calculated by the comparative CT (2^-*ΔΔ*Ct^) method. mRNA expression levels in the normal control group were used as a calibrator. Values are expressed as the mean ± SEM. *n* = 8 for each group at 12, 24, and 48 h after cecal ligation and puncture (CLP). Differences among groups at each time point were analyzed by a one-way ANOVA followed by Tukey's post-hoc test. The symbol ^#^ significantly differs from the SH group; the symbol “^∗^” significantly differs from the SS group (*p* < 0.05).

Compared to the sham group, the macrophage infiltration markers, CD68 and EMR-1, and inflammatory cytokines, IL-1*β*, IL-6, and TNF-*α*, were all elevated after CLP. These indicators were significantly lower in the SG group than those in the SS group at each time point, except that CD68 and EMR-1 at 48 h post-CLP which showed no differences between the two sepsis groups (Figure 5).

### 3.6. Tight Junction, Macrophage Infiltration, and Antiapoptotic Gene Expressions in the Lungs in Obese Mice with Sepsis

**Figure 6:**
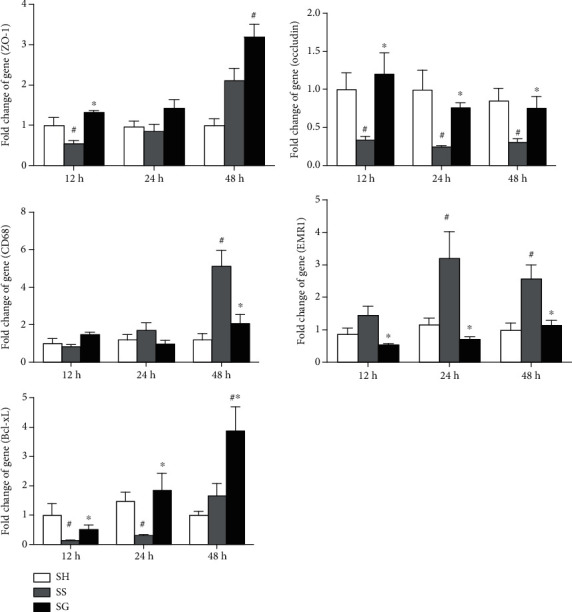
Expressions of genes related to macrophage infiltration, tissue injury, and antiapoptosis in the lungs. Cluster of differentiation 68 (CD68), epidermal growth factor-like module-containing mucin-like hormone receptor-like (EMR)-1, zonula occludens (ZO)-1, and antiapoptotic marker B cell lymphoma-extra large (Bcl-xL). SH: sham-operated group; SS: sepsis group with saline; SG: sepsis group with glutamine. mRNA changes were quantitated and analyzed by a real-time PCR and calculated by the comparative CT (2^-*ΔΔ*Ct^) method. mRNA expression levels in the normal control group were used as a calibrator. Data are shown as the mean ± SEM. *n* = 8 for each group at 12, 24, or 48 h after cecal ligation and puncture (CLP). Differences among groups at each time point were analyzed by a one-way ANOVA followed by Tukey's post-hoc test. The symbol “^#^” significantly differs from the SH group; the symbol “^∗^” significantly differs from the SS group (*p* < 0.05).

As to tight junction genes, obesity with sepsis exhibited lower ZO-1 gene expression at 12 h, while occludin expression levels were lower at 12, 24, and 48 h after CLP compared to those of the SH group. Sepsis with the GLN administration exhibited higher tight junction gene expressions than the SS group at those time points. The expression of the macrophage infiltration marker, CD68, was significantly higher in the SS group at 48 h, whereas the EMR-1 expression was higher at 24 and 48 h after CLP. Obese mice with the GLN administration after sepsis showed significantly lower CD68 at 48 h and EMR-1 at 24 and 48 h after CLP. There were no differences in CD68 or EMR-1 expressions between the SG and SH groups at each time point. The expression of the antiapoptotic gene, Bcl-xL, was lower in the SS group at 12 and 24 h compared to the SH group after CLP. Compared to the SS group, the SG group had higher Bcl-xL expression at each time point post-CLP (Figure 6).

## 4. Discussion

In this study, we created a mouse model of obesity complicated with sepsis, because currently, the concomitant presence of obesity and sepsis is an important cause of hospital admissions and mortality in critically ill patients [22, 23]. GLN was intravenously administered immediately after CLP, and a second dose was provided 24 h after the first injection. A previous study reported that a single dose of GLN resulted in an increment in plasma GLN levels within 15 min [24], which alleviated sepsis-associated organ damage and improved survival post-CLP [25, 26]. A booster dose might enhance the efficacy of GLN used in the body during catabolic conditions. We did not include lean septic mice with/without GLN, because the impacts of GLN on lean mice with polymicrobial peritonitis had been investigated previously in our laboratory [13–15]. The focus of this study was the comorbidity of obesity and sepsis. This model can possibly be applied to abdominal surgical obese patients with infections. We analyzed the subpopulation distributions of Th cells including Th1/Th2/Th17 and Treg cells in the circulation. Also, markers of systemic and local inflammation were measured. Since the lungs are the most frequently affected remote organ in sepsis, markers of apoptosis and lung injury were evaluated. We found that underlying obesity exaggerated inflammation and organ injury due to sepsis. The GLN administration after sepsis exhibited a more balanced ratio of Th/Treg cells and alleviated adipocyte inflammation and remote lung injury.

Th cells differentiate into different subsets which produce distinct cytokines in response to the infection [18]. Th1 cells enhance IFN-*γ* and IL-2 production during intracellular infections [27], while Th2 cells produce IL-4, IL-5, and IL-13 to promote humoral immunity [28]. Th17 cells produce IL-17, IL-22, and TNF-*α* in response to the bacterial pathogen invasion [29]. Treg cells are another kind of Th cell that act against the Th17 and suppress excessive T cell responses [30]. A previous study found that obesity results in increased Th2/Th17 phenotypes and decreased Treg cells [2]. The results of this study showed that obesity with sepsis exhibited more pronounced percentages of IL-4- and IL-17-expressing Th cells than did obesity alone. A previous study found that the balance of Th subsets changing toward the Th2 and Th17 response may aggravate inflammation and contribute to sepsis progression [31]. Consistent with the polarization toward the Th2/Th17 lineages, leptin and inflammatory cytokine expressions in plasma, PLF, and adipose tissues were upregulated with the concurrence of obesity and sepsis. A study by Kolyva et al. also reported that obesity in the presence of sepsis is associated with increased proinflammatory cytokine production and exaggerated systemic oxidative stress [23].

In addition to inflammatory cytokines, we also analyzed several markers in this study. CD68 is a protein highly expressed by macrophages in inflamed tissues [32]. EMR-1, also known as F4/80, is a well-known and widely used marker of murine macrophages [33]. Macrophage infiltration into enlarged adipocytes results in persistent proinflammatory mediator production that leads to systemic inflammation in obesity [34]. In this study, CD68 and EMR-1 expressions in the SS group were elevated, suggesting that sepsis concomitant with obesity did increase macrophage infiltration and exaggerate inflammation of adipose tissues. Our findings are in agreement with a previous report that macrophages increased in visceral adipose tissues when sepsis occurred in obese subjects [23]. In order to evaluate injury to the lungs, tight junctions were analyzed. Tight junctions are proteins that play important roles in forming a barrier against extracellular substances and acting as transporters between cells [35]. Occludin is a transmembrane protein, and ZO-1 is a cytoplasmic protein which interacts with occludin and is involved in the intracellular signaling [36]. Occludin and ZO-1 are highly expressed in various organs, including the lungs [35]. Occludin functions as an air-blood barrier in the lungs [37]. Apoptosis is a form of cell death controlled by the intracellular caspase family. Bcl-xL is the most abundant Bcl-xL protein that functions to inhibit cell apoptosis [38]. Results of this study revealed lower antiapoptotic and tight junction gene expressions in the SS group, indicating that sepsis-associated lung injury and apoptosis were occurring in obesity complicated with sepsis.

In this study, we administered GLN to obese mice after CLP and found several effects that were not observed in mice with a saline injection. First, the GLN administration reduced Th2/Th17 and Treg percentages in the circulation. This may attenuate sepsis-induced systemic inflammation and prevent immunosuppression. A previous study also found that the GLN administration elicited a more balanced CD4 T cell polarization [14] and downregulated programmed death-1 expression by T cells, thus preventing immunosuppression during sepsis [15]. Second, inflammatory mediators, including plasma leptin and IL-1*β* and IL-6 concentrations in PLF, were reduced. Also, expressions of macrophage infiltration markers, including CD68 and EMR-1, in adipose tissues and the lungs decreased, indicating that local and systemic inflammation was attenuated by the GLN administration. Third, the tight junction-associated compounds, ZO-1 and occludin, and antiapoptotic gene expressions in lung tissues were significantly higher in the GLN-administered group, suggesting that sepsis-associated cell apoptosis and tissue injury were attenuated. Previous studies also showed that supplemental GLN prevented *γδ* T cell apoptosis and neutrophil infiltration to the lungs and thus alleviated remote lung damage during sepsis [14, 20].

A previous multicenter clinical trial reported that GLN supplementation is detrimental to critically ill patients [39]. Since GLN absorption and utilization may be impaired in patients with multiple organ failure, the outcomes mentioned above might not be extrapolated to all septic conditions. Actually, subsequent clinical studies found that GLN is safe and had favorable effects in stabilized patients with organ function and resolution of shock [40, 41]. Possible explanations of the favorable effects of the GLN administration observed here may involve several mechanisms. GLN is an important fuel source for immune cells and is essential for T cell proliferation and activation [42]. GLN supplementation after sepsis may provide a source of fuel for effector T cell activation to fulfill body needs and maintain a more balanced Th cell polarization. A previous study revealed that GLN inhibited the nuclear factor (NF)-*κ*B activation, which led to suppression of its downstream target gene expressions, thus reducing the production of inflammatory mediators [43]. A recent in vitro study also reported that the administration of GLN reduced expressions of proinflammatory genes and proteins by adipocytes [44]. On the other hand, GLN is the precursor of the endogenous antioxidant, glutathione. Since inflammation and oxidative stress aggravate tissue injury, the lower inflammatory mediator production and GLN-associated redox reactions may be partly responsible for alleviating organ injury. Both obesity and sepsis are syndromes associated with oxidative stress and metabolic disorders. Obesity complicated with sepsis would amplify inflammation and immune dysregulation. However, elucidating the mechanisms of how GLN acts on regulating T cell polarization and inflammatory response underlying the coexistence of obesity and sepsis requires further investigation.

To the best of our knowledge, this is the first study to investigate the effects of GLN on T cell immune regulation in obesity complicated with sepsis. Results showed that the coexistence of obesity and sepsis aggravated inflammation and dysregulation of CD4 T cells beyond that exerted by obesity alone. With the underlying obesity, the GLN administration after CLP attenuated local and systemic inflammation, modulated a more balanced Th polarization, and alleviated remote lung injury associated with sepsis. The findings presented here provide basic information and imply that GLN may have a potential to regulate T cell homeostasis, attenuate inflammation, and provide protective effects for obese patients at risk of abdominal sepsis.

## Figures and Tables

**Table 1:** Composition of the high-fat diet.

Ingredient	g/kg
Casein	259.13
*L*-Cysteine	3.89
Maltodextrin	161.96
Sucrose	89.14
Cellulose	64.78
Soybean oil	32.39
Lard	317.44
Mineral mix^(1)^	12.96
Dicalcium phosphate	16.84
Calcium carbonate, 1H_2_O	7.13
Potassium citrate	21.38
Vitamin mix^(2)^	12.96
Total	1000

^(1)^The composition of the mineral mixture is listed as follows (mg/g): calcium phosphate dibasic, 500; sodium chloride, 74; potassium sulfate, 52; magnesium oxide, 24; potassium citrate monohydrate, 20; manganese carbonate, 3.5; ferric citrate, 6; chromium potassium sulfate, 0.55; zinc carbonate, 1.6; curpric carbonate, 0.3; potassium iodate, 0.01; and sodium selenite, 0.01. ^(2)^The composition of the vitamin mixture is listed as follows (mg/g): DL-*α*-tocopherol acetate, 20; nicotinic acid, 3; retinyl palmitate, 1.6; calcium pantothenate, 1.6; pyridoxine hydrochloride, 0.7; thiamin hydrochloride, 0.6; riboflavin, 0.6; cholecalciferol, 0.25; D-biotin, 0.05; menaquinone, 0.005; and cyanocobalamin, 0.001.

**Table 2:** Sequences of oligonucleotide primers used for PCR amplification.

		Primer sequences (5′⟶3′)
GAPDH	Forward	AACGACCCCTTCATTGAC
	Reverse	TCCACGACATACTCAGCAC
TNF-*α*	Forward	ATGGCCTCCCTCTCATCAGT
	Reverse	TTTGCTACGACGTGGGCTAC
IL-1*β*	Forward	TGCCACCTTTTGACAGTGATG
	Reverse	ATGTGCTGCTGCGAGATTTG
IL-6	Forward	TCCTACCCCAACTTCCAATGCTC
	Reverse	TTGGATGGTCTTGGTCCTTAGCC
CD68	Forward	TGTTCAGCTCCAAGCCCAAA
	Reverse	ACTCGGGCTCTGATGTAGGT
EMR-1	Forward	ACCTTGTGGTCCTAACTCAGTC
	Reverse	ACAAAGCCTGGTTGACAGGTA
ZO-1	Forward	GGCACATCAGCACGATTTCT
	Reverse	CCACAAAAGAAATCCTTTCACACCT
Occludin	Forward	ACTGGGTCAGGGAATATCCA
	Reverse	TCAGCAGCAGCCATGTACTC
Bcl-xL	Forward	ACCTCCTCCCCGACCTATGA
	Reverse	CTATCTCCGGCGACCAGCAA

GAPDH: glyceraldehyde 3-phosphate dehydrogenase; TNF: tumor necrosis factor; IL: interleukin; CD68: cluster of differentiation 68; EMR-1: epidermal growth factor-like module-containing mucin-like hormone receptor-like 1; ZO-1: zonula occludens, Bcl-xL: B cell lymphoma-extra large.

## Data Availability

The data used to support the findings of this study are included within the article.
